# The ACHRU-CPP versus usual care for older adults with type-2 diabetes and multiple chronic conditions and their family caregivers: study protocol for a randomized controlled trial

**DOI:** 10.1186/s13063-017-1795-9

**Published:** 2017-02-06

**Authors:** Maureen Markle-Reid, Jenny Ploeg, Kimberly D. Fraser, Kathryn Ann Fisher, Noori Akhtar-Danesh, Amy Bartholomew, Amiram Gafni, Andrea Gruneir, Sandra P. Hirst, Sharon Kaasalainen, Caralyn Kelly Stradiotto, John Miklavcic, Carlos Rojas-Fernandez, Cheryl A. Sadowski, Lehana Thabane, Jean A. C. Triscott, Ross Upshur

**Affiliations:** 1 Aging, Community and Health Research Unit (ACHRU), School of Nursing, McMaster University, 1280 Main Street West, Hamilton, ON L8S 4K1 Canada; 2 Faculty of Nursing, University of Alberta, 11405-87 Avenue, Edmonton, AB T6G 1C9 Canada; 3 School of Nursing, McMaster University, 1280 Main Street West, Hamilton, ON L8S 4K1 Canada; 4 Department of Clinical Epidemiology and Biostatistics, Centre for Health Economics and Policy Analysis, McMaster University, 1280 Main Street, Hamilton, ON L8S 4K1 Canada; 5 Department of Family Medicine, University of Alberta, 6-40 University Terrace, Edmonton, AB T6G 2T4 Canada; 6 Faculty of Nursing, University of Calgary, 2500 University Drive NW, Calgary, AB T2N 1N4 Canada; 7 Department of Family Medicine, McMaster School of Medicine, Principal, CRF Consulting, 763 Cedar Bend Drive, Waterloo, ON N2V 2R6 Canada; 8 Faculty of Pharmacy and Pharmaceutical Sciences, University of Alberta, 3-229 Edmonton Clinic Health Academy, 11405 87 Avenue, Edmonton, AB T6G 1C9 Canada; 9 Department of Clinical Epidemiology and Biostatistics, St. Joseph’s Healthcare Hamilton, Room H-325, 50 Charlton Avenue East, Hamilton, ON L8N 4A6 Canada; 10 Care of the Elderly Division, Department of Family Medicine, University of Alberta, Edmonton, AB T6G 2R7 Canada; 11 Division of Clinical Public Health, Dalla Lana School of Public Health, University of Toronto, 155 College Street, 6th floor, Toronto, ON M5T 3M7 Canada

**Keywords:** Type-2 diabetes mellitus, Randomized controlled trial, Pragmatic, Hybrid effectiveness-implementation design, Self-management, Group-based programs, Community-based settings, Health-related quality of life, Older adults

## Abstract

**Background:**

Many community-based self-management programs have been developed for older adults with type-2 diabetes mellitus (T2DM), bolstered by evidence from randomized controlled trials (RCTs) that T2DM can be prevented and managed through lifestyle modifications. However, the evidence for their effectiveness is contradictory and weakened by reliance on single-group designs and/or small samples. Additionally, older adults with multiple chronic conditions (MCC) are often excluded because of recruiting and retention challenges. This paper presents a protocol for a two-armed, multisite, pragmatic, mixed-methods RCT examining the effectiveness and implementation of the Aging, Community and Health Research Unit-Community Partnership Program (ACHRU-CPP), a new 6-month interprofessional, nurse-led program to promote self-management in older adults (aged 65 years or older) with T2DM and MCC and support their caregivers (including family and friends).

**Methods/design:**

The study will enroll 160 participants in two Canadian provinces, Ontario and Alberta. Participants will be randomly assigned to the control (usual care) or program study arm. The program will be delivered by registered nurses (RNs) and registered dietitians (RDs) from participating diabetes education centers (Ontario) or primary care networks (Alberta) and program coordinators from partnering community-based organizations. The 6-month program includes three in-home visits, monthly group sessions, monthly team meetings for providers, and nurse-led care coordination. The primary outcome is the change in physical functioning as measured by the Physical Component Summary (PCS-12) score from the short form-12v2 health survey (SF-12). Secondary client outcomes include changes in mental functioning, depressive symptoms, anxiety, and self-efficacy. Caregiver outcomes include health-related quality of life and depressive symptoms. The study includes a comparison of health care service costs for the intervention and control groups, and a subgroup analysis to determine which clients benefit the most from the program. Descriptive and qualitative data will be collected to examine implementation of the program and effects on interprofessional/team collaboration.

**Discussion:**

This study will provide evidence of the effectiveness of a community-based self-management program for a complex target population. By studying both implementation and effectiveness, we hope to improve the uptake of the program within the existing community-based structures, and reduce the research-to-practice gap.

**Trial registration:**

ClinicalTrials.gov, Identifier: NCT02158741. Registered on 3 June 2014.

**Electronic supplementary material:**

The online version of this article (doi:10.1186/s13063-017-1795-9) contains supplementary material, which is available to authorized users.

## Background

The prevalence of diabetes is increasing in countries across the globe, particularly type-2 diabetes mellitus (T2DM) [1]. Currently, 347 million people have diabetes worldwide and this is expected to increase by 55% by 2035 [1, 2]. T2DM comprises 90% of all diabetes cases [3] and results from genetic, behavioral, and environmental risk factors [1, 4]. Advancing age is associated with T2DM, with older adults having the highest prevalence of T2DM of any age group [1, 5]. Among older adults, T2DM frequently occurs in conjunction with other comorbidities [1, 6, 7]. Studies suggest that upwards of 40% or more people with T2DM have three or more comorbid health conditions [8–10]. When considered within the context of other comorbidities, T2DM represents a significant burden in older adults that is linked to higher mortality, reduced functional status, increased use of health care services, and higher risk of institutionalization [5, 11]. Thus, there is an urgent requirement to provide services that meet the needs of this complex population.

There is strong evidence that the primary determinants of T2DM, notably smoking, poor diet, obesity, and physical inactivity are modifiable through self-management activities [2, 12–17]. From large clinical trials, such as the American Diabetes Prevention Program (DPP) [18] and the Finnish Diabetes Prevention Study (FDPS) [19, 20], there is evidence that lifestyle and weight-loss programs can reduce the incidence of diabetes by up to 58%. The benefits can be enduring [4]; one study reported a 34% reduction in incidence rates among its intervention group during a 10-year follow-up [21] and a 4-year study reported sustained weight loss and improved cardiovascular risk factors following intensive lifestyle intervention [22]. Thus, aggressive public policy initiatives have been recommended to encourage self-management of lifestyle change [23]. Strong support for these initiatives can be found in a recent systematic review showing that integrated care interventions for people with T2DM (which target self-management and patient-centered, team-based approaches to care) can result in improvements in a range of clinical and patient-reported outcomes, with no studies reporting a worsening in outcomes [24].

Clinic-based lifestyle interventions like the DPP and FDPS require substantial resources (e.g., specialized expertise, individual settings); thus, researchers have assessed whether interventions delivered in community settings might be an effective, economical, and feasible alternative. Recent international studies, for example, have reported that group-based diabetes programs, established in community settings, result in improved clinical, lifestyle, and psychosocial outcomes compared to routine care [25–27]. Importantly, community-based programs have proven to be scalable and sustainable at a national level [28] and they are beneficial to individuals of many ages, including older adults [29–31]. In a systematic review involving 16 studies that translated the DPP into hospital, primary care, community, and work settings Whittemore [32] concluded that programs linked to existing structures of care, (e.g., the Young Men’s Christian Association, or YMCA) may enhance adoption, implementation, and maintenance of the program.

While there is strong evidence for self-management interventions in general, the existing research, particularly for community-based interventions, has limitations. Notably, evidence for their effectiveness is equivocal. Whittemore [32] concluded that self-management interventions delivered in community settings, despite their ability to reach diverse populations, reported less weight loss than those interventions offered in more restricted settings, such as hospitals. In contrast, studies by Ali et al. [33] and Ruggiero et al. [34] found that interventions with community components (e.g., lay members or community health workers) resulted in more weight loss compared to those run by medical or allied health professionals. A different challenge relates to validity and reliability since many effectiveness studies of community-based interventions rely on single-group designs and/or pilot studies with small samples [35]. Additionally, comparatively few studies have examined the effectiveness of lifestyle interventions in older adults with T2DM and comorbid conditions, because this population is typically excluded from randomized controlled trials (RCTs) [5, 36]. With their increased risk of comorbidity and geriatric syndromes, such as cognitive impairment, falls, and depression [37–40], this medically complex population is typically more difficult to reach, recruit, and retain [7]. Thus, there is uncertainty about the clinical effectiveness of self-management interventions for older adults with multiple chronic conditions (MCC). More information is also needed on adapting community-based interventions to individual settings, the effectiveness of interventions in key patient subgroups, and operational costs.

**Fig. 1:**
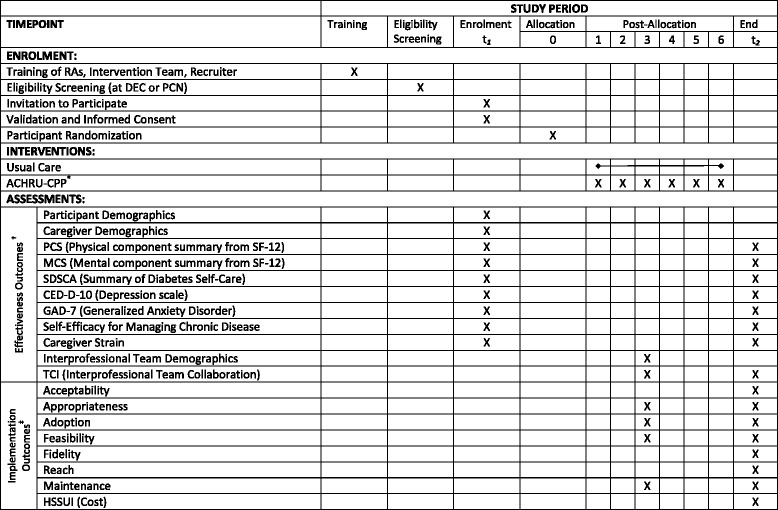
Standard Protocol Items: Recommendations for Interventional Trials (SPIRIT) Checklist: schedule of enrollment, interventions, and assessments

The purpose of this article is to describe a study designed to examine the effectiveness and implementation of the Aging, Community and Health Research Unit-Community Partnership Program (ACHRU-CPP), a new 6-month interprofessional, nurse-led program to promote self-management in older adults (aged 65 years and older) with T2DM and MCC and their family caregivers (including family and friends). The description follows the Standard Protocol Items: Recommendations for Interventional Trials (SPIRIT) guidelines [41], which provide a list of the recommended items to include in clinical trial protocols. The populated SPIRIT Checklist is available in Additional file 1. The trial is a multisite, two-arm, pragmatic, mixed-methods RCT that employs a type-2 hybrid design which simultaneously evaluates both clinical effectiveness (e.g., Does the intervention work?) and implementation (e.g., Is the intervention delivery feasible and acceptable?) [41]. The participant timeline recommended by SPIRIT [41] shows the schedule of enrollment, interventions, and assessments (see Fig. 1) and these are all discussed in greater detail below. Hybrid designs are thought to facilitate the transition from research to practice and result in more rapid uptake of effective interventions [42]. The trial is based on a pilot study, where the program’s feasibility and potential effectiveness were demonstrated [43].

### Objectives

The aim of the trial is to evaluate the clinical effectiveness and implementation of the ACHRU-CPP, compared to usual care. We hypothesize that clients in the intervention group will experience greater improvements in health-related quality of life (HRQoL) compared to the usual care group. We also expect that the program will improve outcomes for family caregivers, improve interprofessional team functioning among the interventionists, and be feasible to implement in practice at no additional cost. The study will address the following specific objectives:

1. To compare the effects of the intervention versus usual care on older adults’ HRQoL, diabetes self-management, self-efficacy, depressive symptoms, anxiety, and the costs of use of health services (from a societal perspective)
2. To determine the subgroups of older adults that benefit most from the program
3. To compare the effects of the intervention versus usual care on family caregivers’ HRQoL, strain, depressive symptoms, and the costs of use of health services (from a societal perspective), and
4. To evaluate the implementation of the program and its effects on interprofessional/team collaboration

## Methods/design

This study is a pragmatic RCT which combines a quantitative analysis of the effects of the intervention with a qualitative and quantitative analysis of the implementation and contextual factors potentially associated with variations in the outcomes in the real world. Combining quantitative and qualitative analyses in an intervention study has been referred to as a concurrent, embedded mixed-methods design in the mixed-methods literature [44] and a hybrid trial in the interventions literature [42]. The choice to include both quantitative and qualitative methods in our study reflects our view that no single method can fully answer the questions posed by an intervention study. While an RCT is regarded as the “gold standard” for establishing effectiveness of interventions, effect sizes do not provide policy-makers with information on how an intervention might be replicated in their specific context, or whether trial outcomes can be reproduced. Guidelines for evaluating complex interventions have been updated to recognize the value of using qualitative research methods to complement the quantitative effects [45].

**Fig. 2:**
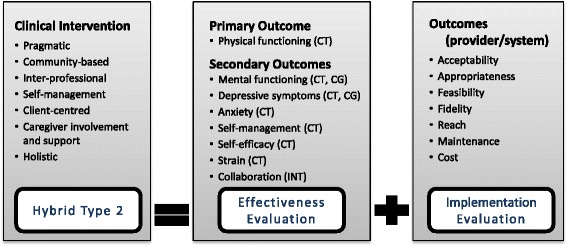
Type-2 hybrid effectiveness – Implementation evaluation framework

Figure 2 provides the overall evaluation framework for the trial. The evaluation of effectiveness and implementation outcomes, while discussed separately below, will occur simultaneously, receive equal emphasis, and are recognized as often dependent on each other (e.g., the effectiveness of the intervention will depend on the quality and success of the implementation strategy and process). Our approach of assigning equal weight to the effectiveness and implementation components of the study is consistent with a type-2 hybrid trial [42]. Implementation outcomes examined in the trial are guided by two published frameworks: the broad implementation research framework by Peters et al. [46] and the Consolidated Framework for Implementation Research (CFIR) for systematically identifying contextual factors that can impact intervention implementation, adoption and maintenance by Damschroder et al. [47]. The Peters et al. [46] framework recognizes a range of implementation outcomes including: acceptability, adoption, appropriateness, feasibility, fidelity, cost, coverage, and sustainability. The CFIR provides a focused set of constructs to evaluate barriers and facilitators including characteristics of the intervention, outer and inner setting, individuals delivering the intervention, and the implementation process. We have developed research questions and quantitative measures and/or qualitative inquiries corresponding to each implementation outcome from these two frameworks.

### Participants and setting

A strategic first step for this study was the development of a partnership between researchers in the Aging, Community and Health Research Unit (ACHRU) at McMaster University (Hamilton, ON, Canada) and the University of Alberta (Edmonton, AB, Canada), program coordinators from seven community-based organizations, and seven diabetes education centers (Ontario) or primary care networks (Alberta). The study will enroll 160 participants in each of two Canadian provinces, Ontario and Alberta. Although these provinces are in the same country, they encompass diverse characteristics in terms of geography (Ontario is in the east, Alberta the west), culture, and health care system structure. In Ontario, study participants will be recruited from clients who have been recently referred for diabetes-related services to diabetes education centers (DECs). DECs are regional centers that provide ongoing diabetes education programs, counselling and follow-up sessions by registered nurses (RNs) and registered dietitians (RDs) for clients and their families. In Alberta, study participants will be recruited from clients who have been recently referred to diabetes or chronic disease management programs at three primary care networks (PCNs). PCNs are centralized resource facilities that provide equipment and health care teams to service clients from physician clinics in close proximity to one another. The Ontario and Alberta provinces were selected to compare and contrast different health care systems. The specific sites within each province were selected because they serve a large and growing older adult population, and demonstrated strong support for the program. Community partner organizations were selected and invited based on existing collaborations with the sites and/or interest in health promotion for community-dwelling older adults. The community partners were also selected based on availability and accessibility of an appropriate space to host monthly group program sessions and availability of staff members to participate in program delivery (e.g., a program coordinator for the group sessions).

In order to participate in the study, clients must satisfy the following inclusion criteria:

- have enrolled in a DEC (Ontario) or referred to a PCN (Alberta) within the past 24 months, or recruited from the community
- 65 years of age or older
- diagnosed with at least two chronic conditions in addition to T2DM
- not planning to move away from the community in the next 6 months, and
- able to speak English (or with an interpreter available)

These inclusion criteria were designed to be minimally stringent in order to facilitate the broad applicability of the results to the general population of community-dwelling older adults with T2DM and MCC. Aspects of specific criteria were also selected to ensure consistency with broader research initiatives.

### Screening for eligibility and enrolment

In Ontario, trained DEC staff will identify potential clients based on the inclusion criteria and then contact them by phone to obtain verbal consent to be contacted by a research assistant (RA). A RA will then conduct an in-home interview to obtain written informed consent and complete the baseline questionnaires. Differing legislation required that study personnel use a different approach in Alberta. First, the recruiter identifies potential clients and then establishes their eligibility and interest. If written consent-to-be-contacted was obtained during recruitment, the client would be contacted by a RA. If there was no written consent-to-be-contacted, then it would be left to the potential client to contact the research team. At the time of recruitment, all eligible clients will be asked to invite their family caregivers (family or friend of at least 18 years of age who provides physical, emotional, or financial care to the client) to participate in the study, and provide their contact information. Participation of a family caregiver is encouraged but not required.

At the initial home visit the RA will first administer the Short Portable Mental Status Questionnaire [48] to assess the client’s mental status. A score of 5 or higher is required for the client to provide consent, and if the client scores below 5, their family caregiver will be invited to consent on behalf of the client. Once the appropriate informed consent has been received, study clients and/or their family caregivers will complete the baseline interview and questionnaires. Interviews and completion of the questionnaires is expected to take about 2 h.

### Program

The RCT is pragmatic, which means that the program will be implemented under real-world conditions, including reliance on existing staff at participating sites. A detailed description of the program is available elsewhere [43]. The program will be delivered by an interprofessional team consisting of RDs and RNs from the participating DECs/PCNs and a program coordinator (PC) from the community partner agencies. As the program is designed to work with the existing personnel within these organizations, there may be additional professionals, such as kinesiologists or pharmacists, who are part of the interprofessional teams. The providers involved in delivering the program in each study site will not provide care to participants randomized to the usual care group. Clients randomly assigned to the control group will continue to be offered usual care services through their local DEC/PCN. The specific services that comprise usual diabetes care vary across both provinces in terms of the length and focus of educational sessions, whether classes are strongly recommended versus optional (e.g., foot care, cardiac health, eating and exercise interventions), access to on-site professionals (e.g., endocrinologist, dietician, physiotherapist, exercise specialist, pharmacist), connections with support services and community resources, and type of follow-up services available.

Clients randomly assigned to the intervention group will be offered the program in addition to the usual care services that they currently receive. The program is a 6-month, multicomponent, client-driven strategy that was designed to support self-management of diabetes and other MCC. It was tested in a pilot study, and was modified based on the feedback received from clients and interventionists [43]. The program consists of the following components: (1) up to three in-home visits by an RN and RD, (2) a monthly group wellness program, hosted by the community partner agency and supported by peer volunteers, (3) monthly case conferences involving the RN, RD, PC, and (3) care coordination and navigation to link clients to other health care professionals and community support services as needed. Because the program is client-driven, there is flexibility in the components in terms of the mode of delivery, specific activities emphasized, and dosage. For example, the client may decline one or more home visits or group sessions, or they may choose the DEC/PCN or alternate setting instead of their home for the visits. A graphical display outlining the intervention versus usual care services is available in Additional file 2 [49].

The fundamental principles underlying the components of the client-driven program are self-efficacy, collaboration, holistic care, and caregiver engagement and support (Table 1). The client is a key member of the care team and is fully engaged in the development of a care plan that is tailored to their individual needs and preferences. The central role of the client is consistent with the current policy priorities of the Ontario [50] and Alberta [51] health ministries. Client self-efficacy, a major determinant of a person’s behavior and motivation to take action, has been found to be a predictor of diabetes self-management [52]. Bandura, the psychologist credited with first recognizing the central role of self-efficacy in behaviour, defined self-efficacy as “the belief in one’s capabilities to organize and execute the courses of action required to manage prospective situations” ([53], p. 2). Key sources of self-efficacy are mastery, social modelling, social persuasion and psychological state such as mood and stress [54]. Various aspects of the program target these sources, including: home visits and group sessions which include education and reinforcement of appropriate self-management principles and activities; group sessions which provide opportunities for social modelling and persuasion; home visits which include motivational interviewing to encourage positive psychological states and celebrate successes by modifying goals and care plans as participants master self-management tasks; and meaningful and inclusive collaboration involving all members of the care team, including clients and their caregivers, to foster their commitment and deeper interest in diabetes self-management.

**Table 1:** Principles underpinning the Aging, Community and Health-Community Partnership Program (ACHRU-CPP)

Component	Feature(s)
Self-efficacy	• strengthening confidence of clients and their family caregivers in their abilities to monitor their health, make decisions, and adopt healthy self-care behaviors
Collaborative practice	• involving all members of the care team (interventionists, clients and family caregivers) in all decisions relating to the program • emphasizing flexibility in responses so that individual client preferences can be effectively met • integrating and sharing knowledge from all members of the care team into decisions • optimizing the scopes of practice of the interventionists (e.g., professionals trained in health promotion and prevention can fully utilize these skills, all team members can participate in care planning)
Holistic care	• working with clients to apply self-management principles to the unique set of chronic conditions and risk factors they face (e.g., income, social supports) • developing a care plan that is realistic in view of the client’s strengths, challenges and preferences • integrating evidence-based practices for diabetes with those relating to the other chronic conditions
Caregiver engagement and support	• inviting caregivers to actively participate in home visits, group sessions, and case conferences • incorporating caregiver insights/feedback into the development of a care plan that best meets the client’s needs • ensuring that support services are provided to caregivers to promote their health and wellbeing and assist them in the caregiving role

### Program implementation strategy

A four-pronged approach will be used by the researchers to implement and monitor the delivery of the program at each site to ensure trial adherence:

- *Educational workshops*: the investigators will hold training sessions for the interventionists and peer support volunteers before implementation of the program. Each session will be supported with role-appropriate training manuals
- *Outreach visits*: the investigators and research coordinators will conduct monthly meetings either in person or by teleconference with the interventionists and peer support volunteers with each of the seven sites to discuss the progress of the study, provide feedback and education, and discuss barriers encountered and possible solutions for identified barriers
- *Reminders*: the investigators and research coordinators will provide updates on the study to the interventionists and managers of the participating DECs/PCNs and community partner agencies, including successes and areas for improvement related to the program
- *Audit and feedback*: the interventionists will be asked to complete forms (i.e., visit reports and team meeting records) related to program-specific activities that were carried out. At 1-month intervals, the investigators and research coordinators will conduct audits of the study-related documentation to assess fidelity to the program (refer to Additional file 3)

### Participant flow, assessments and timeline

**Fig. 3:**
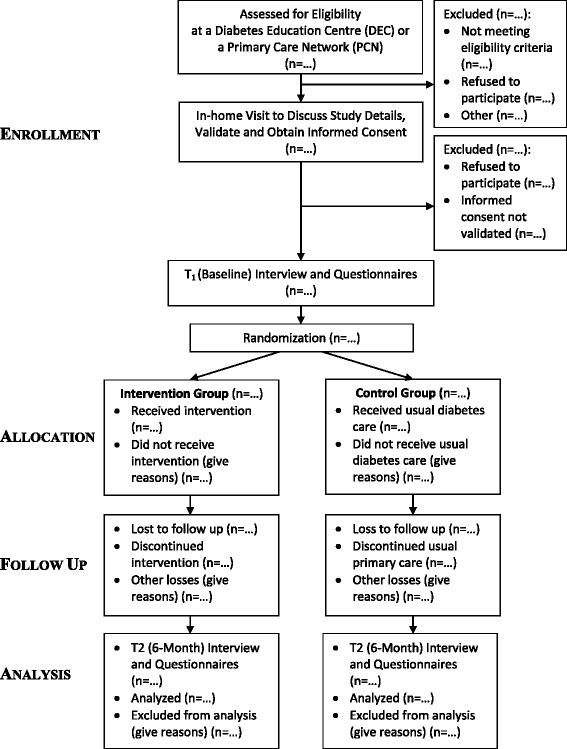
Flow diagram of progress through study phases

The study involves two assessments: one at baseline (*T*_1_) and the other 6 months after baseline (*T*_2_). At baseline, data will be collected on demographic, clinical, and socioeconomic variables in addition to assessment of the primary and secondary outcome variables. The flow of participants through the study phases will be presented using a flow diagram which conforms to the Consolidated Standards of Reporting Trials (CONSORT) guidelines [55] for pragmatic trial reporting (Fig. 3).

### Sample size

The sample size for the RCT was calculated to detect a minimally important difference (MID) in the primary outcome measure (PCS-12). The developers suggest that the MID for interpretation of group mean PCS-12 score differences is approximately 3.0, which corresponds to an effect size of 0.30 [56]. However, the developers also acknowledge that this MID estimate is a matter of ongoing debate. Robust research on effect sizes seen across a range of studies using a variety of quality-of-life measures has shown that the mean effect size is remarkably consistent at 0.50, or half the standard deviation [57]. This effect size is close to the one observed in our pilot study, which was 0.40 [43].

We based our estimate of the sample size on an effect size of 0.50, 5% alpha, 80% power and 20% attrition (18% was observed in our pilot study). Using these assumptions, the sample size was 80 (each) for the intervention and control groups.

### Randomization design

After providing written, informed consent and completing the baseline questionnaires, participants will be randomly assigned to either the intervention or the control arm of the study using a 1:1 allocation ratio. Stratified permuted block randomization will be used to assign participants to the control and intervention groups. Study sites are expected to be relatively homogeneous regarding client sociodemographic and clinical characteristics and the delivery of intervention and usual care services. However, to minimize the confounding effects of possible differences among the study sites, the sites will be used as a stratum. The randomized sequence at each site will be based on assignments of blocks randomly selected from sizes of 2, 4, or 6. A biostatistician not involved in the recruitment process will generate the random number assignments for each site using SAS Version 9.3. Random number sequences will be input into a centralized web-based randomization service (REDCap) that will allocate clients to the control or intervention group at each site in accordance with the sequence.

### Blinding

After randomization, the interventionists and clients will know the group assignments. This lack of blinding is unavoidable; however, efforts will be made to blind the assessors who conduct the 6-month assessment. Some unblinding may occur, for example, if a client describes a home visit or group session in their interview with the assessor, even if such information is not requested. The statistician/data analyst will be blinded to the group assignments.

#### Analysis of clinical effectiveness

##### Outcome measures

Table 2 describes the primary and secondary outcome measures used to evaluate clinical effectiveness. The primary outcome is the change in Physical Component Summary (PCS-12) score from the short form-12v2 health survey (SF-12) [58]. The SF-12 is a generic quality-of-life instrument that is well-validated and able to distinguish between groups of clients with known clinical differences in a variety of populations [58]. The pilot study supported the selection of the PCS-12 score as the primary outcome by demonstrating that the PCS-12 was responsive over the 6-month intervention period and grounded in questions that were easily interpreted by the older adult clients [43]. The PCS-12 is also a common primary outcome in studies evaluating programs similar to ours [59] and focuses on an outcome (physical functional ability) that is important to clients [60]. Secondary outcomes include the change in the following variables from baseline (*T*_1_) to 6 months (*T*_2_): Mental Component Summary (MCS) score from SF-12 [61]; Center for Epidemiological Studies Depression Scale (CES-D-10) [62–65]; Generalized Anxiety Disorder 7-item scale (GAD-7) [66–70]; Summary of Diabetes Self Care Activities (SDSCA) [71, 72]; and the Modified Caregiver Strain Index (CSI) [73].

**Table 2:** Effectiveness outcomes, target population, and analysis

Effectiveness outcomes				
Variable/outcome	Hypothesis	Measure	Group(s)^a^	Method of analysis
Physical functioning	Improve more in the intervention group	Physical Component Summary (PCS-12) [58]	CT, CG	Means and standard deviations, *t* tests for comparing change from baseline (*T*_1_) to 6 months (*T*_2_) in the intervention and control groups ANCOVA analysis to explore predictors of primary outcome multiple regression with backward selection to explore impact of dose on primary outcome
Mental functioning	Improve more in the intervention group	Mental Component Summary (MCS) from SF-12 [61]	CT, CG	
Depressive symptoms	Larger reduction in the intervention group	Center for Epidemiological Studies Depression Scale (CES-D-10) [62–65]	CT, CG	
Anxiety	Larger reduction in the intervention group	Generalized Anxiety Disorder 7-item Scale (GAD-7) [66–70]	CT	
Self-management	More improvement in the intervention group	Summary of Diabetes Self Care Activities (SDSCA) [71, 72]	CT	
Self-efficacy	More improvement in the intervention group	Self-Efficacy for Managing Chronic Disease 6-item scale [52, 74–81]	CT	
Caregiver strain	Larger reduction in the intervention group	Modified Caregiver Strain Index (CSI) – 13 items [73]	CG	
Interprofessional team collaboration	Improvement in collaboration over 6 months in the intervention group	Team Climate Inventory (TCI) [82–84]	INT	

Demographic data will be collected from all participant groups. All clients will be asked to provide their age, gender, education, and diabetes history (duration, family history). Clients and family caregivers will also be asked to provide household income, marital status, and comorbid health conditions. Caregivers will be asked to provide their employment status and ethnic back ground. Interventionists will be asked about the number of years working in their field and the number of years in their current role

^a^Three groups: *CT* client, *CG* caregiver, *INT* interventionists (e.g., RN, RD, PC)

To better understand the mechanisms underlying the changes in primary and secondary outcomes, changes from baseline (*T*_1_) to 6 months (*T*_2_) in the following measures will be assessed: (1) Self-Efficacy for Managing Chronic Disease 6-item scale [52, 74–81]; items in the scale are common across many chronic diseases, including symptom control, role function, emotional functioning and communicating with physicians, and (2) Team Climate Inventory (TCI) [82–84] (19-item); that includes participative safety (trust in group members), support for innovation (openness to new ideas), vision (shared goals and valued outcomes), and task orientation (shared concern for excellence) is evaluated.

### Analyses

#### Analysis of participants

The data collected will be screened for accuracy, missing data, outliers, and statistical assumptions. SAS Version 9.3 will be used for all statistical analyses. All statistical tests will be performed using two-sided tests at the 0.05 significance level. For all models, the results will be expressed as effects, standard errors, 95% confidence intervals, and associated *p* values. The evaluation will be conducted in accordance with the intention-to-treat principle; therefore, imputation (using multiple imputation) will be used to address missing data and compare these results with the complete case analysis. If there are discrepancies between the multiple imputation and complete case results, we will conduct a sensitivity analysis using different multiple imputation methods (appropriate for the pattern of missingness) to see how robust the analysis is for the chosen method(s) of handling missing data [85]. This procedure will result in multiple data sets and models being run on each data set. Appropriate methods will be used to combine the results to generate pooled parameter estimates, standard errors, and confidence limits.

An analysis of baseline (*T*1) data will be performed to compare the demographic and clinical characteristics of the intervention and control groups. This analysis will confirm whether the two groups are equal on these variables. Means and standard deviations will be generated for continuous variables, and frequencies and proportions for categorical variables. The appropriate significance test (e.g., *t* test for continuous measures, chi-square for categorical) will be applied to identify any significant differences between the groups. Significant differences will be adjusted for in the outcomes analyses.

For the analyses relating to clients, *t* tests will be used to compare the changes in primary and secondary outcomes from baseline (*T*_1_) and 6 months (*T*_2_) in the two groups. Primary and secondary measures will also be analyzed using repeated measures ANCOVA (two time periods) to understand the factors shaping these outcomes. Site will be included as a predictor, consistent with our randomization strategy which stratified by site. Other potential covariates include age, gender, self-efficacy, duration of diabetes, and level of comorbidity. Covariates will be identified and selected for inclusion in the models based on bivariate analyses to confirm their relationship with the outcome and the absence of a significant relationship with other covariates. We will conduct a cross-jurisdictional comparison across Ontario and Alberta, given the differences in community-based structures.

We will also examine the relationship between the dose of the intervention and the primary outcome variable (6-month PCS-12 score) in order to better understand the mechanisms underlying this outcome and to tease out quantity versus quality effects. Regression will be used and dose will be measured by the number of home visits and number of group sessions attended (explored as individual components and as a combined measure).

The effectiveness of an intervention is often dependent on the characteristics of the population; thus, this study will include a subgroup analysis to identify what clients benefit most from which approach to treatment [86]. A range of characteristics thought to influence self-management behavior will be evaluated, including: age, gender, duration of diabetes, depressive symptoms, number of comorbidities, self-efficacy and caregiver support. Regression using two-way interactions between the study group (intervention versus control) and each characteristic will be conducted to examine subgroup effects [87, 88]. The models will use the PCS-12 score for the dependent variable and independent variables will include group assignment, client characteristic (e.g., at least three versus less than three comorbidities), and the interaction term.

Regarding the family caregivers, data on their demographic characteristics and the three outcomes (HRQoL, depressive symptoms, and strain) will be collected at baseline and again at 6 months. Descriptive statistics will be generated to summarize the characteristics and outcomes for the family caregivers in each group, with *t* tests and chi-square tests being used to evaluate group differences in continuous and categorical variables, respectively.

Statistical procedures for interventionist outcomes are limited by the small number of individuals involved; therefore, descriptive analysis will be mainly used to report the results. We anticipate collecting 21 TCI assessments (baseline and 6 months), one for each of three members of the program team (RN, RD, PC) at each of the sites. We will attempt to explore potential site differences and relationships with key variables (e.g., dose of the intervention, primary and secondary outcomes of T2DM clients).

#### Analysis of implementation

##### Outcomes

Table 3 provides the implementation outcomes and related measures for evaluating the implementation of the program. Implementation outcomes include acceptability, appropriateness, adoption, feasibility, fidelity, reach (coverage), maintenance (sustainability), and cost. Acceptability will be assessed using the enrollment, attrition, and engagement rates (percentage of study participants receiving at least one home visit and attending at least one group session), and dose. Targets have been set for each of these measures based on the medical literature and/or our pilot study results [43], and observed values from the RCT will be compared to the targets. The following targets have been set for the RCT: enrollment rate of >50%, attrition rate of <20%, engagement rate for home visits >90% and group sessions >75%, and a median dose of three or more in-home visits and attendance at group sessions.

**Table 3:** Implementation outcomes – Measures and method of analysis

Implementation outcomes		
Outcome	Outcome measure(s)	Methods of analysis
*Acceptability*: a willingness to receive the offered intervention	- Enrollment rate (%) - Attrition/retention rate (%) - Engagement rate (% at least 1 home visit and 1 group session) - Dose of the intervention (number of visits and/or group sessions attended)	- Compare observed rates to targets set for study - Characteristics of consenters versus nonconsenters - Descriptive statistics for total dose, number of visits, number of group sessions
*Appropriateness:* from the perspective of interventionists	- Perceived benefit to participants - Convenience of implementation	Qualitative descriptive analysis of focus group and outreach meetings
*Adoption:* the intervention is appealing to providers and realistic to implement in practice	- Enrollment rate (%) - Attrition/retention rate (%) - Engagement rate (% at least 1 home visit and 1 group session) - Dose of the intervention (number of visits and/or group sessions attended)	- Compare observed rates to targets set for study - Characteristics of consenters versus nonconsenters - Descriptive statistics for total dose, number of visits, number of group sessions
*Feasibility*: the capability to carry out intervention activities	- Training of the interventionists - Delivery of the program - Perceptions of barriers and facilitators	- Evaluation of training materials, log sheets - Qualitative descriptive method to analyze focus group content
*Fidelity*: adherence to intervention components	Refer to Fidelity Checklist (Additional file 3)	- Review records of attendance at training session and monthly meetings with researchers - Review home visit records and log sheets to assess delivery of the program as planned
*Reach*: degree to which the target population is eligible to receive the intervention (coverage)	- Proportion of clients eligible - Reasons for exclusion - Characteristics of eligible clients	- Descriptive statistics
*Maintenance*: the extent to which the intervention can be sustained	- Engagement rate (%) - Attrition /retention (%) - Reported reasons for attrition - Prospects for the program’s uptake in practice setting	- Descriptive statistics - Qualitative descriptive method to analyze focus group content
*Cost*: from a societal perspective	- Health and Social Services Utilization Inventory (HSSUI) [90]	- Comparison of baseline (*T*_1_) and 6-month (*T*_2_) median costs using Mann-Whitney *U* test

Appropriateness and feasibility will be evaluated based on the results of the monthly outreach meetings and focus group sessions with interventionists. These meetings and focus group sessions will provide information on the perceived impact, and barriers and facilitators to implementation of the program. Questions asked during the focus group session will be guided by the CFIR [47] framework which facilitates the systematic exploration of the appropriateness and feasibility of the program in terms of barriers and facilitators. CFIR focuses on examining the following key domains: intervention characteristics (e.g., complexity of intervention, perception of benefits and relative advantage compared to usual practice), outer setting (e.g., credibility of intervention by provider and senior administrative agents), inner setting (e.g., team characteristics and engagement, level of coordination and collaboration, involvement of clients and caregivers, compatibility with existing systems and resources), and individuals (e.g., enthusiasm and support for intervention, consistent tracking of activities, reporting and resolving challenges, robust referrals).

Costing will assume a societal perspective [89], which implies collecting all costs, regardless of who bears them. The wider the perspective taken, the more applicable the study is to social-policy decisions. The costs of use of all types of health services from baseline to 6 months in T2DM clients and their family caregivers will be determined using the Health and Social Services Utilization Inventory (HSSUI) [90]. The HSSUI consists of questions about the respondent’s use of the following direct health care services: (1) primary care, (2) emergency department and specialists, (3) hospital days, (4) other health and community support services, and (5) prescription medications. Services specific to the program will be captured such as interventionist training, delivery of services in group sessions and in-home visits, and attendance at case conferences and outreach visits. Inquiries are restricted to the reliable duration of recall: 6 months for remembering a hospitalization and a visit to the physician, and 2 days for use of a medication. The HSSUI builds on the work of Browne et al. [91, 92] whose work was tested and assessed for reliability and validity and was acknowledged as one of the few published measures of ambulatory utilization that is empirically validated [93]. The 6-month cost data will be derived from “quantity” data for the services identified in the HSSUI and current “price” data for each service [90]. Services include: physician costs, home care services, outpatient costs, X-rays, other health care providers, emergency services, medication costs, tests, emergency room and hospitalization, and caregiver support services [90]. The product of the number of units of service (quantity) and unit cost (price) is total cost.

### Implementation analyses

Table 3 also provides the types of analyses that will be undertaken in the implementation evaluation. Both quantitative and qualitative methods will be employed in the evaluation, depending on the outcome being examined. Acceptability will be assessed by comparing observed rates (e.g., enrollment, eligibility, engagement, and dose) with preset targets. Many of these rates will also be used in evaluating maintenance. Reach will be assessed by reviewing baseline characteristics of the total sample and comparing these to the broader population of older adults with T2DM and MCC. A review of the records on eligible clients will also be conducted, to compare those that enroll in the study to those that do not enroll, withdraw or are lost. These records will capture demographic characteristics (e.g., age, gender, comorbidity, and site) as well as the reasons clients declined to participate, withdrew, or were lost. These analyses will enable us to assess the representativeness of the intervention group compared to those that were eligible but declined to participate, withdrew, or were lost. We will also examine the reasons for refusal and evaluate whether declines can be attributed to the nature of the program being offered.

Qualitative descriptive methods will be used to assess the content of focus groups, outreach meeting records, and other documents (e.g., log sheets documenting delivery of program components and principles used). Data from the focus groups will be digitally recorded and transcribed by an experienced transcriptionist and checked for accuracy by a RA. The data will be managed using N-Vivo 10 software. Content analysis will be used to analyze the transcripts. Two research investigators will independently review all transcripts and inductively generate a list of codes describing the content. The codes will then be grouped into themes (a higher conceptual level) and subthemes. Difference of opinion between the two investigators will be discussed until agreement is reached.

The cost analyses will involve comparing the cost of use of specific health and social services between the intervention and control groups. Because cost data are often right skewed, we anticipate using nonparametric tests (e.g., Mann-Whitney *U* test) to evaluate differences in median costs between the two groups. We will also estimate the costs of the program, and total costs of health care and social service in the two groups.

## Discussion

This paper describes the design of a multisite, two-arm, pragmatic, mixed-methods RCT of the effectiveness of the ACHRU-CPP aimed at improving self-management in older adults with T2DM and MCC and providing support to caregivers. This research makes several important contributions to the existing knowledge base. First, it investigates the effectiveness of a self-management intervention in a complex population. This population is particularly at risk of adverse outcomes, yet they are often excluded from RCTs. As a result, their needs are poorly understood and evidence of the effectiveness of interventions aimed at behavioral change in this vulnerable group is lacking. The exclusion of this group is important; about 60% of older adults with T2DM have at least one comorbid condition and 40% have three or more [8–10]. Second, the study includes a subgroup analysis to determine which study clients benefit most from the program. Few intervention studies conduct this type of analysis, yet it is critical for informing the implementation of the program in other settings and to target scarce resources to those most likely to benefit [86]. Third, the study includes a cost analysis, which provides policy-makers with critical information on the resource implications of the program to facilitate decision-making.

There are several strengths of the design of this study. First, the intervention being evaluated targets behavioral change, consistent with the epidemiological roots of T2DM and supported by behavioral theory and empirical results demonstrating the effectiveness, sustainability and scalability of self-management interventions for T2DM. Second, the study combines effectiveness and implementation research, which is consistent with a comprehensive approach to evaluation and offers the potential to reduce the research-to-practice gap [42]. With equal weighting assigned to both implementation and effectiveness, our study best fits the type-2 hybrid effectiveness-implementation study design using the typology proposed by Curran et al. [42]. These researchers argue that hybrid designs offer the potential to accelerate the translation of research into routine practice, compared to the more dominant approach of proceeding in step-wise fashion (beginning with clinical efficacy, then clinical effectiveness and finally implementation research). Third, our study is designed to be rich in information, providing a considerable amount of data covering a range of outcomes (e.g., self-care, mental health, self-efficacy, strain, collaboration) for multiple stakeholders (e.g., clients, family caregivers, health care providers). Fourth, we have chosen objective measures for the outcomes examined, using well-established and validated instruments. Finally, our study involves multiple sites and jurisdictions, which will enhance the generalizability of the study’s findings.

There are some challenges that should also be acknowledged. The study is examining the immediate 6-month effects of the program, which represents the early stages of implementation. While this timeframe was sufficient to demonstrate the feasibility and potential effectiveness in the pilot study [43], it limits our ability to comment on the long-term sustainability of the program. We will not be able to forecast long-term behavioral change in clients, nor will we be able to comment on the program’s sustainability within the partner organizations after the study concludes. During the interviews and focus groups, we will ask interventionists about their intentions to gain insight into the potential for sustainability. Notably, the community partners who participated in the pilot demonstrated “buy-in” and a commitment to ongoing planning, implementation, and evaluation of the program [43]. Adoption and implementation has the potential to be high given that the program involves the combination of diabetes expertise and increased accessibility because it is community-based.

Self-management interventions are also recognized to be challenging to implement when they are delivered in diverse community-based settings and feature a plan of care that is individualized, sustainable, and targeted to a complex population. The program is reliant on having resources available within the community, and smaller or rural regions might not have the sufficient infrastructure to offer the program (or all aspects of it). It can also be difficult to reach community-dwelling older adults with T2DM and MCC. Recruiting targets typically set for younger demographics may not be realistic for this population [94]. Retention may also be difficult; researchers have reported high attrition rates in older populations [95, 96]. In an effort to address these challenges, the study team will strengthen training and communication with RAs. By using these mechanisms, recruitment can be improved because interviewers will be trained to build rapport and trust, while they also rely on clear guidelines for participant contact, flexible interview schedules that accommodate client preferences and information that appropriately orients participants to research including its procedures, risks, and benefits. Importantly, participants will be given adequate time to decide whether to participate. Similarly, retention will be enhanced because interventionists will be guided to maintain regular and personalized contact with participants for the duration of the study. The research team will also facilitate retention by mailing reminder letters at 2 months and having the RA make a reminder phone call at 4 months. The researchers will also meet regularly with the interventionists to proactively address any issues related to the delivery of the program.

Finally, we have designed the program to be delivered in two different jurisdictions and multiple sites. The strength of this approach is that it will increase the generalizability of the results, potentially improve the uptake of results in new communities, and speed the translation of research into practice. However, involving multiple sites also introduces certain challenges. As this is a pragmatic trial, we both expect and encourage interventionists to make adaptations to address their local context and client needs. The risk is that these adaptations may lead to variability in the program’s delivery, particularly in component features and dose. Close monitoring will help the investigators to evaluate fidelity, or the consistency with which the components were applied, as well as the dose of the intervention actually delivered [97]. We will examine the impact of this variation on outcomes, similar to a dose-response analysis and address this in our findings [97, 98]. We will track any adaptations that are made to the program during its implementation. This information will be used to guide future refinements and prepare for a future study to test the program on a larger scale.

Ultimately, the study results will inform policy concerning community-based interventions to enhance self-management of T2DM. The study includes outcomes for a range of stakeholders (clients, family caregivers, health care providers) and includes a cost analysis. It will inform both clients and health care providers and improve our understanding of the effectiveness of group-based interventions in changing self-management behaviors in a complex and underserved population (older adults with T2DM and MCC). By including the evaluation of both effectiveness and implementation in one study design, we hope to enhance the relevance and sustainability of the study results to clinicians and policy-makers, and reduce the research-practice gap.

## Additional files

Additional file 1: ACHRU-CPP: SPIRIT Checklist. (PDF 96 kb)
Additional file 2: ACHRU-CPP: Graphical depiction of intervention compared to control group. (PDF 83 kb)
Additional file 3: ACHRU-CPP: Fidelity Checklist. (PDF 87 kb)

## Abbreviations

ACHRU: Aging, Community and Health Research Unit (McMaster University)
ACHRU-CPP: Aging, Community and Health Research Unit-Community Partnership Program
CES-D-10: Center for Epidemiological Studies on Depression (10 items)
CFIR: Consolidated Framework for Implementation Research
CSI: Modified Caregiver Strain Index
DEC: Diabetes Education Center
DPP: Diabetes Prevention Program
FDPS: Finnish Diabetes Prevention Study
GAD-7: Generalized Anxiety Disorder (7 items)
HiREB: Hamilton Integrated Research Ethics Board
HRQoL: Health-related quality of life
HSSUI: Health and Social Services Utilization Inventory
MCC: Multiple chronic conditions
MCS-12: Mental Component Summary Score (SF-12)
MID: Minimally important difference
PC: Program coordinator
PCN: Primary Care Network
PCS-12: Physical Component Summary Score (SF-12)
RA: Research assistant
RCT: Randomized control trial
RD: Registered dietitian
RN: Registered nurse
SDSCA: Summary of Diabetes Self Care Activities
SF-12: Short-Form Health Survey (12 items)
T2DM: Type-2 diabetes mellitus
TCI: Team Climate Inventory
YMCA: Young Men’s Christian Association
