# Supplementary material for: The ACHRU-CPP versus usual care for older adults with type-2 diabetes and multiple chronic conditions and their family caregivers: study protocol for a randomized controlled trial
Source: Trials. 2017 Feb 6;18:55. doi: 10.1186/s13063-017-1795-9 (PMC5294729; doi:10.1186/s13063-017-1795-9)
Supplement: Additional file 1: — ACHRU-CPP: SPIRIT Checklist. (PDF 96 kb) [file 13063_2017_1795_MOESM1_ESM.pdf]

SPIRIT 2013 Checklist: Recommended items to address in a clinical trial protocol and related documents*

Section/item	Item No	Description	Addressed on page number
**Administrative information**			
Title	1	Descriptive title identifying the study design, population, interventions, and, if applicable, trial acronym	1
Trial registration	2a	Trial identifier and registry name. If not yet registered, name of intended registry	3
	2b	All items from the World Health Organization Trial Registration Data Set	-
Protocol version	3	Date and version identifier	Dec. 2 2014, Version 3
Funding	4	Sources and types of financial, material, and other support	28
Roles and responsibilities	5a	Names, affiliations, and roles of protocol contributors	1-2; 29
	5b	Name and contact information for the trial sponsor	No sponsor
	5c	Role of study sponsor and funders, if any, in study design; collection, management, analysis, and interpretation of data; writing of the report; and the decision to submit the report for publication, including whether they will have ultimate authority over any of these activities	28

	5d	Composition, roles, and responsibilities of the coordinating centre, steering committee, endpoint adjudication committee, data management team, and other individuals or groups overseeing the trial, if applicable (see Item 21a for data monitoring committee)	See footnote[1]
**Introduction**			
Background and rationale	6a	Description of research question and justification for undertaking the trial, including summary of relevant studies (published and unpublished) examining benefits and harms for each intervention	4-7
	6b	Explanation for choice of comparators	10
Objectives	7	Specific objectives or hypotheses	6-7
Trial design	8	Description of trial design including type of trial (eg, parallel group, crossover, factorial, single group), allocation ratio, and framework (eg, superiority, equivalence, noninferiority, exploratory)	7; 14
**Methods: Participants, interventions, and outcomes**			
Study setting	9	Description of study settings (eg, community clinic, academic hospital) and list of countries where data will be collected. Reference to where list of study sites can be obtained	8-9; 29
Eligibility criteria	10	Inclusion and exclusion criteria for participants. If applicable, eligibility criteria for study centres and individuals who will perform the interventions (eg, surgeons, psychotherapists)	9-10
Interventions	11a	Interventions for each group with sufficient detail to allow replication, including how and when they will be administered	10-12; Additional file 2

[1] The coordinating centre is located at the Aging, Community and Health Research Unit (ACHRU), McMaster University and it is responsible for study oversight and data management. The Scientific Advisory Committee of ACHRU will provide input on all aspects of the study including implementing the research plan, translating the findings into policy and practice, and disseminating the findings.

	11b	Criteria for discontinuing or modifying allocated interventions for a given trial participant (eg, drug dose change in response to harms, participant request, or improving/worsening disease)	See footnote[2]
	11c	Strategies to improve adherence to intervention protocols, and any procedures for monitoring adherence (eg, drug tablet return, laboratory tests)	13; 19; 25
	11d	Relevant concomitant care and interventions that are permitted or prohibited during the trial	10-11
Outcomes	12	Primary, secondary, and other outcomes, including the specific measurement variable (eg, systolic blood pressure), analysis metric (eg, change from baseline, final value, time to event), method of aggregation (eg, median, proportion), and time point for each outcome. Explanation of the clinical relevance of chosen efficacy and harm outcomes is strongly recommended	15-17; 19-21
Participant timeline	13	Time schedule of enrolment, interventions (including any run-ins and washouts), assessments, and visits for participants. A schematic diagram is highly recommended (see Figure)	13; Figure 2; Table 2; Additional file 2
Sample size	14	Estimated number of participants needed to achieve study objectives and how it was determined, including clinical and statistical assumptions supporting any sample size calculations	14
Recruitment	15	Strategies for achieving adequate participant enrolment to reach target sample size	9-10; 25

Methods: Assignment of interventions (for controlled trials)

Allocation:

Sequence generation	16a	Method of generating the allocation sequence (eg, computer-generated random numbers), and list of any factors for stratification. To reduce predictability of a random sequence, details of any planned restriction (eg, blocking) should be provided in a separate document that is unavailable to those who enrol participants or assign interventions	14-15

[2] During study interviews, if a study participant shows any signs of emotional distress due to the questions that are being asked and does not want to continue, the interview will be terminated and appropriate assistance offered to the participant through their local diabetes education centre. Nurses and dietitians conducting home visits will notify the participant's family physician and/or make referrals to other members of the health care team to address concerns raised during home visits. Participants will be encouraged to limit the suggested home-support exercises to what feels comfortable to them. Interventionists will be trained to develop a supportive environment within the group sessions for peer sharing and learning.

Allocation concealment mechanism	16b	Mechanism of implementing the allocation sequence (eg, central telephone; sequentially numbered, opaque, sealed envelopes), describing any steps to conceal the sequence until interventions are assigned	14-15
Implementation	16c	Who will generate the allocation sequence, who will enrol participants, and who will assign participants to interventions	14-15
Blinding (masking)	17a	Who will be blinded after assignment to interventions (eg, trial participants, care providers, outcome assessors, data analysts), and how	15
	17b	If blinded, circumstances under which unblinding is permissible, and procedure for revealing a participant's allocated intervention during the trial	15
**Methods: Data collection, management, and analysis**			
Data collection methods	18a	Plans for assessment and collection of outcome, baseline, and other trial data, including any related processes to promote data quality (eg, duplicate measurements, training of assessors) and a description of study instruments (eg, questionnaires, laboratory tests) along with their reliability and validity, if known. Reference to where data collection forms can be found, if not in the protocol	Table 2; Table 3; 15-16; 19-21
	18b	Plans to promote participant retention and complete follow-up, including list of any outcome data to be collected for participants who discontinue or deviate from intervention protocols	17
Data management	19	Plans for data entry, coding, security, and storage, including any related processes to promote data quality (eg, double data entry; range checks for data values). Reference to where details of data management procedures can be found, if not in the protocol	See footnote[3]

[3] Data will be collected by Research Assistants (RAs) via paper data collection forms (DCFs) or by an online data collection format (LimeSurvey). Data entered manually by RAs will be transcribed into electronic format and verified by two research coordinators (RCs). Data entered directly in an electronic data collection format will be verified by one RC. In both cases, as data are received, the RC will resolve any quality and accuracy issues with RAs. Access to data files and research records will be restricted to authorized study personnel. Security measures will be implemented to ensure that data are securely transmitted and stored. Digital data files will be encrypted and password protected, fax devices will be situated in secure locations and hard copy formed will be stored in locked cabinets. Confidentiality measures will be implemented, specifically: documents will be labeled with codes, not personal identifiers and the list that links individuals to the codes will be maintained in a separate and secure location. Pseudonyms will be used in reporting study findings.

Statistical methods	20a	Statistical methods for analysing primary and secondary outcomes. Reference to where other details of the statistical analysis plan can be found, if not in the protocol	17-19; Tables 2 & 3; 22-23
	20b	Methods for any additional analyses (eg, subgroup and adjusted analyses)	17-18
	20c	Definition of analysis population relating to protocol non-adherence (eg, as randomised analysis), and any statistical methods to handle missing data (eg, multiple imputation)	17
**Methods: Monitoring**			
Data monitoring	21a	Composition of data monitoring committee (DMC); summary of its role and reporting structure; statement of whether it is independent from the sponsor and competing interests; and reference to where further details about its charter can be found, if not in the protocol. Alternatively, an explanation of why a DMC is not needed	See footnote[4]
	21b	Description of any interim analyses and stopping guidelines, including who will have access to these interim results and make the final decision to terminate the trial	See footnote[5]
Harms	22	Plans for collecting, assessing, reporting, and managing solicited and spontaneously reported adverse events and other unintended effects of trial interventions or trial conduct	See footnote[6]
Auditing	23	Frequency and procedures for auditing trial conduct, if any, and whether the process will be independent from investigators and the sponsor	See footnote[7]

[4] The principal investigator (PI) will assume primary responsibility for monitoring all aspects of the study. The research coordinator (RC) will assist the PI with supervising the recruiter and interviewers, and monitor the progress of the intervention and data collection. Any issues related to fidelity to treatment will be addressed by the PI and RC through monthly outreach meetings with interventionists. The RC will also be responsible for data entry, scrutinizing questionnaires for missing or questionable responses

[5] As this is a low risk intervention, there are no specific interim analysis plans or associated stopping guidelines in the study protocol. Adverse events are reported to the Research Ethics Boards (REB) and if study discontinuation is required we would comply. Also see item 11b above.

[6] Adverse events and unintended effects are documented by the RC. The principal investigators are responsible for assessment and reporting to the applicable Research Ethics Boards. Follow-up will be as determined by the REB.

[7] Monthly outreach meetings between researchers and interventionists will occur to monitor and ensure fidelity of program delivery. The investigators conduct the auditing (no independent auditors).

Ethics and dissemination

Research ethics approval	24	Plans for seeking research ethics committee/institutional review board (REC/IRB) approval	23
Protocol amendments	25	Plans for communicating important protocol modifications (eg, changes to eligibility criteria, outcomes, analyses) to relevant parties (eg, investigators, REC/IRBs, trial participants, trial registries, journals, regulators)	See footnote[8]
Consent or assent	26a	Who will obtain informed consent or assent from potential trial participants or authorised surrogates, and how (see Item 32)	9-10
	26b	Additional consent provisions for collection and use of participant data and biological specimens in ancillary studies, if applicable	n/a
Confidentiality	27	How personal information about potential and enrolled participants will be collected, shared, and maintained in order to protect confidentiality before, during, and after the trial	See 19 above
Declaration of interests	28	Financial and other competing interests for principal investigators for the overall trial and each study site	28
Access to data	29	Statement of who will have access to the final trial dataset, and disclosure of contractual agreements that limit such access for investigators	28
Ancillary and post-trial care	30	Provisions, if any, for ancillary and post-trial care, and for compensation to those who suffer harm from trial participation	-
Dissemination policy	31a	Plans for investigators and sponsor to communicate trial results to participants, healthcare professionals, the public, and other relevant groups (eg, via publication, reporting in results databases, or other data sharing arrangements), including any publication restrictions	See footnote[9]

[8] Research coordinators based in Alberta and Ontario have the responsibility for communicating important protocol modifications. A variety of communication methods (direct contact; resubmission as required; and distribution of revised documents) will be used, as required

[9] Trial results will be shared in academic conferences and in peer-reviewed journals. To promote participant and stakeholder engagement, the investigators will also communicate results using multi-media techniques and at knowledge translation events.

	31b	Authorship eligibility guidelines and any intended use of professional writers	See footnote[10]
	31c	Plans, if any, for granting public access to the full protocol, participant-level dataset, and statistical code	See 29 above
**Appendices**			
Informed consent materials	32	Model consent form and other related documentation given to participants and authorised surrogates	See footnote[11]
Biological specimens	33	Plans for collection, laboratory evaluation, and storage of biological specimens for genetic or molecular analysis in the current trial and for future use in ancillary studies, if applicable	n/a

*It is strongly recommended that this checklist be read in conjunction with the SPIRIT 2013 Explanation & Elaboration for important clarification on the items. Amendments to the protocol should be tracked and dated. The SPIRIT checklist is copyrighted by the SPIRIT Group under the Creative Commons "Attribution-NonCommercial-NoDerivs 3.0 Unported" license.

[10] ACHRU has developed an authorship policy which is available upon request.
[11] Copies of consent forms given to participants are available upon request.
