# Supplementary material for: The ACHRU-CPP versus usual care for older adults with type-2 diabetes and multiple chronic conditions and their family caregivers: study protocol for a randomized controlled trial
Source: Trials. 2017 Feb 6;18:55. doi: 10.1186/s13063-017-1795-9 (PMC5294729; doi:10.1186/s13063-017-1795-9)
Supplement: Additional file 2: — ACHRU-CPP: Graphical depiction of intervention compared to control group. (PDF 83 kb) [file 13063_2017_1795_MOESM2_ESM.pdf]

Graphical Depiction of Intervention Compared to Control Group

Timeline	Intervention	Control<br>Usual Care
Training	A B C	
Recruitment	D	D
Baseline (T_1)	E	E
Randomization	F	F
1 Month	G H I J	H
2 Months	H I J	H
3 Months	G H I J	H
4 Months	H I J	H
5 Months	G H I J	H
6 Months	H I J	H
6 Month (T_2)	K	K

A	Training of the Research Assistants (RA)
B	Training of the Intervention Team
C	Training of Recruiter
D	Recruiter identifies interested and eligible clients
E	RA conducts in-home interviews to explain, screen, consent and administer baseline survey. The family caregiver is invited to complete a questionnaire and return in person or by mail.
F	Randomization
G	In-home or clinic visit [maximum of 3 and minimum of 1, tailored to client needs]
H	Usual diabetes care
I	Group wellness session [maximum of 6 and minimum of 1, tailored to group needs]
J	Monthly case-conference [client care plan and progress review, group wellness session planning]
K	RA administers 6-month survey for clients and family caregivers.

LEGEND: ▢ Fixed Component ◯ Flexible Component (adapted from Perera et al 2007)[49]
