# Supplementary material for: The ACHRU-CPP versus usual care for older adults with type-2 diabetes and multiple chronic conditions and their family caregivers: study protocol for a randomized controlled trial
Source: Trials. 2017 Feb 6;18:55. doi: 10.1186/s13063-017-1795-9 (PMC5294729; doi:10.1186/s13063-017-1795-9)
Supplement: Additional file 3: — ACHRU-CPP: Fidelity Checklist. (PDF 87 kb) [file 13063_2017_1795_MOESM3_ESM.pdf]

Fidelity Checklist

**Program Components**	**Data Source(s)**
Staffing and Supervision	
• Interventionists receive training prior to starting the program<br>• Interventionists meet with investigators on a monthly basis<br>• RAs receive training prior to interviews and administering questionnaires	Record of attendance
Home Visits	
• Initial home visit conducted by the RN & RD<br>• Follow up interim visit by RN or RD<br>• Follow up visit by the RN or RD at 5 months	Provider Log sheet
• Screening for diabetes-related complications and comorbidities<br>• Review of medications	Home Visit Record
• Assessment of self-management of T2DM<br>• Use of motivational interviewing and problem-solving to foster behavioural change and encourage self-care	Home Visit Record
Monthly Group Sessions	
• Participation in the program	Provider Log sheet
• Content of the program	Program Documentation
• Provision of transportation (if needed)	Coordinator's notes
Care Coordination	
• RN helps clients access necessary supports and services in the community as needed	Client Care-plan
Monthly Case Conferences	
• Collaboration and coordination of program components	Team Meeting Record
• Case conference discussions help the intervention team develop and implement a client-centred care-plan for the group program	Client Care-plan
